# Supplementary material for: A Global Screen for Assembly State Changes of the Mitotic Proteome by SEC-SWATH-MS
Source: Cell Syst. 2020 Feb 26;10(2):133–155.e6. doi: 10.1016/j.cels.2020.01.001 (PMC7042714; doi:10.1016/j.cels.2020.01.001)

Q8WUW1 | BRK1_HUMAN | BRK1 C3orf10 HSPC300 MDS027
Monomer MW [kDa]: 8.745 Monomer expected elution fraction: 61

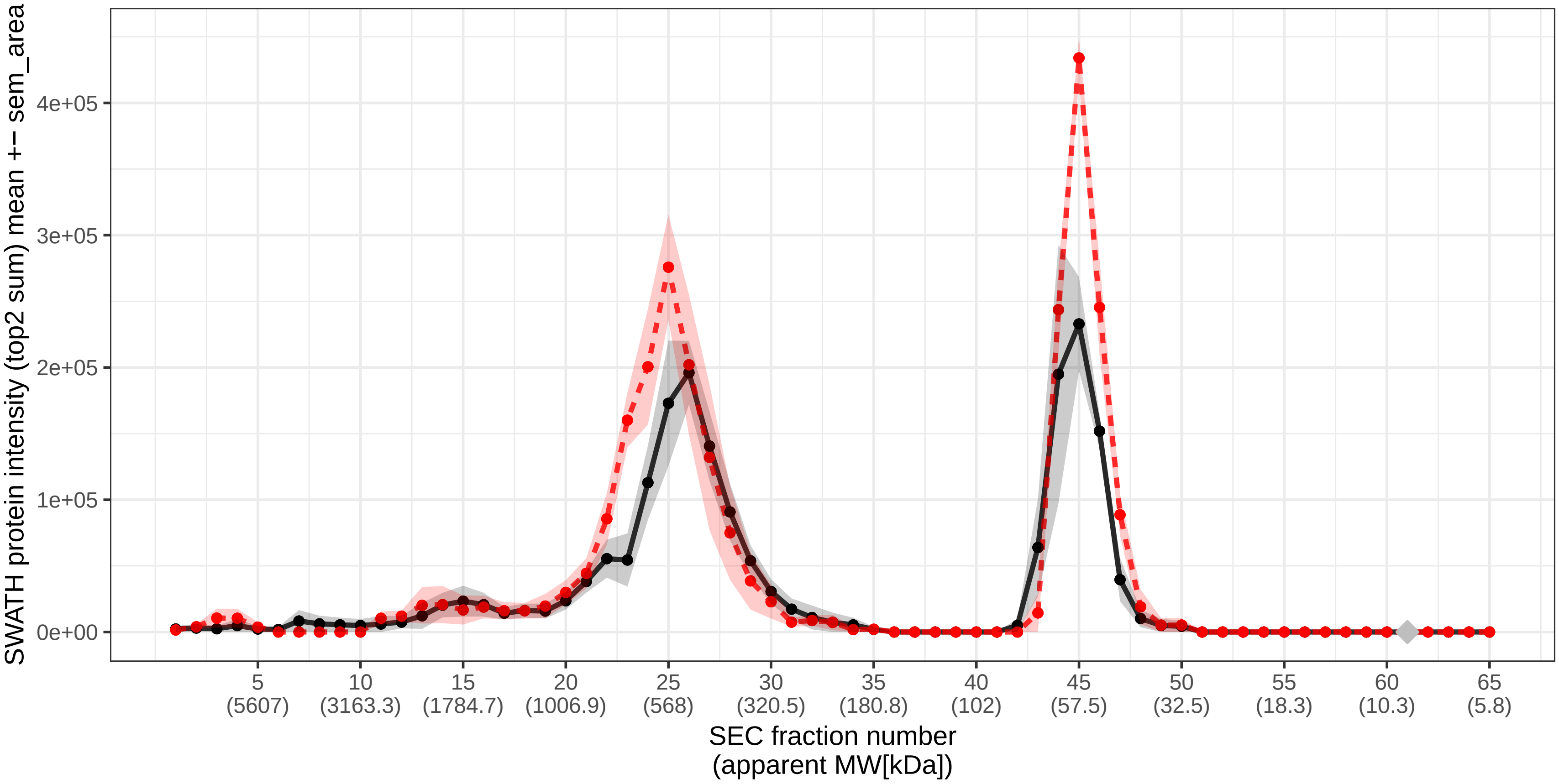

Supplement: Data S1. SEC-SWATH-MS Protein Chromatograms, Related to Figure 1 [file mmc6.zip › SECchrom_Q8WUW1_BRK1_HUMAN_BRK1_C3orf10_HSPC300_MDS027.pdf]
